# Neuronal basis of high frequency fMRI fluctuation: direct evidence from simultaneous recording

**DOI:** 10.3389/fnhum.2024.1501310

**Published:** 2024-10-31

**Authors:** Yang Qiao, Hanbing Lu, Yihong Yang, Yufeng Zang

**Affiliations:** ^1^Centre for Cognitive and Brain Sciences, University of Macau, Macao SAR, China; ^2^Faculty of Health Sciences, University of Macau, Macao SAR, China; ^3^Centre for Cognition and Brain Disorders/Department of Neurology, The Affiliated Hospital of Hangzhou Normal University, Hangzhou, Zhejiang, China; ^4^Institute of Psychological Sciences, Hangzhou Normal University, Hangzhou, Zhejiang, China; ^5^Zhejiang Key Laboratory for Research in Assessment of Cognitive Impairments, Hangzhou, Zhejiang, China; ^6^Neuroimaging Research Branch, National Institute on Drug Abuse, National Institutes of Health, Baltimore, MA, United States; ^7^TMS Center, Deqing Hospital of Hangzhou Normal University, Huzhou, Zhejiang, China; ^8^Collaborative Innovation Center of Hebei Province for Mechanism, Diagnosis and Treatment of Neuropsychiatric disease, Hebei Medical University, Shijiazhuang, Hebei, China

**Keywords:** resting state fMRI, local field potential, simultaneous recording, drug manipulation, frequency-dependent pattern

## Abstract

Resting-state functional magnetic resonance imaging (RS-fMRI) has been extensively utilized for noninvasive investigation of human brain activity. While studies employing simultaneous recordings of fMRI and electrophysiology have established a connection between the low-frequency fluctuation (< 0.1 Hz) observed in RS-fMRI and the local field potential (LFP), it remains unclear whether the RS-fMRI signal exhibits frequency-dependent modulation, which is a well-documented phenomenon in LFP. The present study concurrently recorded resting-state functional magnetic resonance imaging (RS-fMRI) and local field potentials (LFP) in the striatum of 8 rats before and after a pharmacological manipulation. We observed a highly similar frequency-dependent pattern of amplitude changes in both RS-fMRI and LFP following the manipulation, specifically an increase in high-frequency band amplitudes accompanied by a decrease in low-frequency band amplitudes. These findings provide direct evidence that the enhanced high-frequency fluctuations and reduced low-frequency fluctuations observed in RS-fMRI may reflect heightened neuronal activity.

## 1 Introduction

Blood oxygenation level dependent (BOLD) functional magnetic resonance imaging (fMRI) has been extensively utilized for mapping human brain function. Resting-state fMRI (RS-fMRI) is a technique that measures brain activity without requiring participants to perform specific tasks, making it particularly useful for populations with difficulty engaging in tasks, such as young children and patients with severe psychiatric conditions (Biswal et al., 1995). To date, thousands of RS-fMRI studies have been published, shedding light on abnormal spontaneous brain activity across a range of neurological and psychiatric disorders (Bandettini, 2012). Despite these advancements, the precise neurophysiological mechanisms underlying RS-fMRI signals remain largely unknown (Logothetis, 2008).

The BOLD signal serves as an indirect marker of neuronal activity. Approximately two decades ago, Logothetis and colleagues conducted a pioneering study involving simultaneous intracortical recordings of fMRI and electrophysiological signals. Their findings indicated that local field potential (LFP) signals were more predictive of BOLD signal fluctuations compared to multiple unit or single unit activity during visual stimulation, suggesting that the BOLD signal primarily reflects synaptic inputs and local processing rather than neuronal outputs (Logothetis et al., 2001). Since this seminal work, numerous studies have explored the relationship between RS-fMRI signals and electrophysiological activity through simultaneous recordings. Most of these studies have investigated correlations between RS-fMRI time series and LFP power fluctuations (Pan et al., 2011; Pan et al., 2013; Shi et al., 2017; Chan et al., 2017; Liu et al., 2018; Jaime et al., 2019). Notably, significant correlations have been observed between low-frequency components of the RS-fMRI signal (typically 0.01–0.08 Hz) and band-limited power (BLP) time series across broad LFP frequency bands (1–100 Hz) (Pan et al., 2011; Pan et al., 2013; Jaime et al., 2019), with evidence that these correlations are modulated by factors such as anesthesia or pharmacological agents like alpha-amino-3-hydroxy-5-methyl-4-isoxazole propionic acid (AMPA) (Jaime et al., 2019).

It is well-established that increases in neuronal activity are accompanied by elevated LFP amplitudes in high-frequency bands and reduced amplitudes in low-frequency bands (Leopold et al., 2003). Most RS-fMRI studies have focused on conventional low-frequency signals (0.01–0.08 Hz), as higher frequency components (≥ 0.08 Hz) are considered more susceptible to physiological noise from sources such as cardiac and respiratory fluctuations (Birn, 2012). Nevertheless, accumulating evidence suggests that higher frequency RS-fMRI signals may carry important physiological (Boubela et al., 2013; Yuan et al., 2014) or pathophysiological information (Malinen et al., 2010; Otti et al., 2013; Hong et al., 2013; Hong et al., 2018; Ries et al., 2019; Wang et al., 2020). Despite these indications, few studies have examined the neuronal significance of higher frequency RS-fMRI components using simultaneous recordings. Thus, it remains critical to explore whether BOLD signals exhibit a similar frequency-dependent modulation as observed in LFP, characterized by decreased low-frequency and increased high-frequency amplitudes with enhanced neuronal activity. In the present study, we sought to demonstrate a highly similar frequency-dependent pattern of RS-fMRI and LFP amplitudes in response to pharmacological manipulation.

## 2 Materials and methods

### 2.1 Subjects

Eight male Sprague-Dawley rats (275–450g; Charles River) were used in the current study. Each rat was implanted with microelectrode arrays and guide cannulas for pharmacological manipulations. The rats were housed individually in a colony room with ad libitum access to water and food, under a 12-hour light/dark cycle. All experiments were conducted during the light phase. The study procedures were approved by the NIDA-IRP Animal Care and Use Committee.

### 2.2 Experimental design

An MRI-compatible linear microelectrode array was implanted to cover the dorsolateral to ventromedial striatum. A guide cannula was also implanted in the VTA. One week after surgery, MRI and electrophysiological recordings were performed to ensure sufficient surgical recovery. Resting-state scans were initially performed as the baseline session. Subsequently, AMPA was infused into the VTA to enhance dopamine release in the nucleus accumbens, a major target of VTA dopamine neurons (Wang et al., 2015). Special care was taken to minimize MRI-induced electrical artifacts in the local field potential (LFP) recordings, and to ensure the AMPA solution was precisely limited to 1 μl.

#### 2.2.1 Survival surgery: intracranial microelectrode array and guide cannula implantation

**FIGURE 1:**
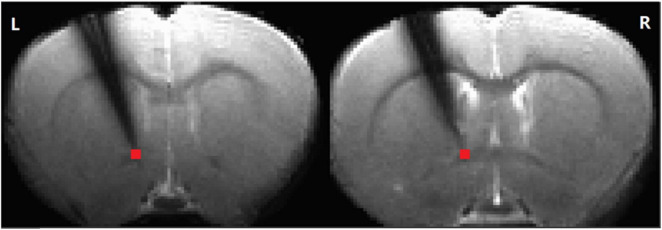
The electrode position. Simultaneous recording of local field potential and resting-state functional magnetic resonance imaging (RS-fMRI) in the striatum of a rat before and after AMPA infusion in the ventral tegmental area (VTA). The time series of the RS-fMRI signal in a region of interest (ROI) at tip (in red, 2 slices, 2 voxels, 0.5 × 0.5 × 1 mm^3^) at the tip of the electrode was extracted for further analysis (L: left. R: right).

To record from the dorsolateral to ventromedial striatal domain, a silicon-based MRI-compatible 16-channel linear electrode array was implanted using aseptic procedures. The electrodes were 15 μm thick, 10 mm long, with 16 gold contacts (30 μm diameter) spaced 200 μm apart (Neuronexus, Ann Arbor, MI) (Figure 1). Rats were initially anesthetized with 2% isoflurane, and their core body temperature, respiration, and arterial oxygenation were continuously monitored and maintained within normal physiological ranges throughout surgery. A 1 mm^2^ craniotomy was made above the nucleus accumbens (NAc) (AP: ++1.2 mm, ML: 3.9 mm, DV: −7.0 mm). The dura was carefully removed using a 27-gauge needle and a #10 scalpel blade. The electrode array was lowered into the brain using a motorized microdriver at a 20° angle (medial-lateral). Dental cement was used to secure the electrode following a 5-minute rest period to allow the tissue to stabilize. A 22-gauge plastic guide cannula (7 mm below pedestal; Plastics One, Roanoke, VA), with a cannula dummy, was implanted into the VTA (AP: −5.0 mm, ML: 0.6–0.8 mm). A ground/reference brass screw, soldered to the ground/reference wires from the electrode using silver solder, was placed over the cerebellum. Rats were given at least one week to recover from surgery before further manipulations. Gentamicin and buprenorphine were administered intraperitoneally to reduce infection risk and alleviate pain during recovery.

#### 2.2.2 fMRI data acquisition

Rats were initially anesthetized with 2% isoflurane until the hind limb reflex was absent. Then, anesthesia was maintained with a continuous loading dose (0.01 mg/kg, i.p.) of dexmedetomidine (Dexdomitor), an α2-adrenergic receptor agonist. Rats were placed in an MRI-compatible holder, and their heads were stabilized using a customized bite bar and ear holders. Body temperature was maintained at approximately 37 ± 0.5°C using a water heating pad and monitored with a rectal temperature probe. A pad was placed between the rat and the heating pad to monitor respiration rate concurrently (SA Instrument Inc., NY, USA). Oxygen saturation was monitored using a noninvasive MRI-compatible pulse oximeter (Starr Life Sciences Corp., PA, USA) attached to a hind paw, with O_2_ saturation maintained at > 96% by adjusting the inhaled oxygen concentration. Isoflurane levels were gradually lowered to 0.5–0.75% depending on the rat’s condition. This anesthesia protocol has been tested in previous studies and shown to have minimal effects on spontaneous brain activity.

MRI data were acquired using a Bruker Biospin 9.4T scanner equipped with an actively shielded gradient coil, running ParaVision 6.0.1 (Bruker Medizintechnik, Karlsruhe, Germany). An 86 mm volume transmitter coil was used for RF excitation, and a 2 cm OD surface coil was placed directly over the skull to encompass the electrode and cannula placement sites for MR signal reception. Tuning and matching of the coils were performed for each subject. T2-weighted anatomical images were acquired using a rapid acquisition with relaxation enhancement (RARE) sequence. Scan parameters were as follows: TR = 2500 ms, TE = 40 ms, RARE factor = 8, field of view (FOV) = 3.5 × 3.5 cm^2^, matrix size = 256 × 256. Functional images were acquired using a gradient echo echo-planar imaging (EPI) sequence with the following parameters: FOV = 3 × 3 cm^2^, matrix size = 64 × 64, TR = 1500 ms, TE = 13 ms, four 1 mm thick slices. The data acquisition bandwidth was 250 kHz. The first three slices covered the striatal region, and the fourth slice was positioned in the VTA. The midsagittal view of the anterior commissure decussation (−0.36 mm from bregma) was used as a fiducial marker to standardize slice localization. fMRI scanning commenced 90 minutes after the dexmedetomidine loading dose, ensuring stable animal physiology and robust BOLD signal (Lu et al., 2012; Jaime et al., 2019; Brynildsen et al., 2016; Hsu et al., 2016). Pre- and post-AMPA infusion scans were conducted sequentially, with each session lasting 7 minutes and 34 seconds, comprising 300 TRs with four dummy scans.

#### 2.2.3 Multiple-channel LFP signal recording concurrent with MRI scan

A customized jumper cable (1.5 feet in length; Plexon Inc., Dallas, TX) was attached directly to the probe implant (Neuronexus, Ann Arbor, MI) when the animal was set well inside the MRI holder. This jumper cable allowed for the headstage to be placed outside of the gradient and RF coils to reduce the MRI- induced artifacts. An MRI compatible headstage (gain = 1) was attached to the jumper cable and the amplifier. A multichannel data acquisition system (Plexon Inc., Dallas, TX) was used for recorded the LFP signals and amplified at a gain of 1000, notch filtered at 60 Hz, and digitally sampled at 5 kHz. Another two separate channels were used to record the respiratory waveform and MRI trigger signal besides the electrode array’s 16 channels. We need to use the trigger signal to remove LFPs’ MRI artifacts. However, in this experiment, we cannot get any recordings at the amplifier for the 15th channel in our system all the time; this channel’s data was abandoned.

#### 2.2.4 Pharmacological manipulation

The striatum is a major projection of the midbrain dopamine neurons. Previous studies had shown that the ventral striatum (nucleus accumbens, NAc) receives projections mainly from the VTA, while the dorsal striatum receives mainly substantia nigra and pars compacta (Ikemoto, 2007). By using the opto-fMRI, recent studies have demonstrated that the dorsal striatum becomes activated in response to optogenetic stimulation of VTA dopaminergic neurons (Beier et al., 2015; Ferenczi et al., 2016; Decot et al., 2017; Lohani et al., 2017). An early study demonstrated that the dopamine release in the ventral striatum could be enhanced by the microinjection of AMPA (RS-alpha-amino-3-hydroxy-5-methyl-4-isoxazolepropionic acid), an ionotropic glutamate receptor agonist (Karreman et al., 1996). Its mechanism is that VTA glutamate neurons synapse on VTA DA neurons and these DA neurons project to the ventral striatum (nucleus accumbens core), and both NMDA and AMPA receptors are involved in this circuit (Wang et al., 2015). This inspired us to manipulate the ventral striatal neuronal activity via similar approach, AMPA injections (Bristol, UK, 1μl, 100μM in saline vehicle) into the VTA.

Specifically, a fused silica needle (Plastics One, Roanoke, VA) was lowered into the implanted guide cannula and connected to a micro-infusion pump via 1.5m long polyethylene tubing (PE 10). We kept the pump outside and the rat inside the scanner on a plane level to minimize solution backflow or leakage. Also, air bubbles inside the infusion line were avoided.

### 2.3 Data processing

In this study, we implanted a guide cannula into the VTA for AMPA infusion and an electrode in the ventral striatum for LFP recording. A total of 12 sessions of simultaneous fMRI and LFP recordings were conducted before and after AMPA infusion across 8 rats. AMPA infusion into the VTA has been well-documented to enhance neuronal activity in the striatum (Jaime et al., 2019; Karreman et al., 1996; Wang et al., 2015). Therefore, we anticipated that both BOLD and LFP signals would exhibit similar patterns of change following the infusion.

#### 2.3.1 LFP data analysis

LFP data analysis was performed using EEGLAB (Delorme and Makeig, 2004) in Matlab (MathWorks, MA, USA). The analysis included MRI artifact removal, filtering, and downsampling. The preprocessed signals were then divided into conventional delta (1–4 Hz), theta (5–8 Hz), alpha (9–14 Hz), beta (15–30 Hz), and gamma (30–50 Hz) frequency bands for subsequent fractional amplitude calculations.

#### 2.3.2 Removal of MRI-induced artifacts

The MR environment induces significant artifacts in LFP signals, which is a common issue in simultaneous recordings. To address this, we applied the following algorithm to remove MR artifacts: LFP signals were segmented using the concurrently recorded trigger signal. Each 60 ms segment immediately following the trigger signal contained high amplitude (up to ± 5 mV) rapidly changing signals, which were replaced by linear interpolation. An eighth-order Butterworth filter was then applied to low-pass filter the resulting signal to 400 Hz using Matlab’s filtfilt() function, which prevents phase nonlinearity and group delays. The filtered data were subsequently downsampled to 1 kHz. The linear interpolation minimized ringing artifacts during filtering. The linearly interpolated segments were further refined using cubic-spline interpolation of the data from 35 ms before and after the artifact segments. Finally, the Butterworth filter and filtfilt() function were applied again to low-pass filter the LFP data to 100 Hz, followed by downsampling to 250 Hz.

#### 2.3.3 LFP fractional amplitude calculation

The preprocessed LFP signals were analyzed using fast Fourier transformation (FFT) for frequencies between 1 Hz and 50 Hz. After denoising, the amplitude of the LFP at the electrode tip channel (Figure 1) was calculated for the following sub-frequency bands: delta (1–4 Hz), theta (5–8 Hz), alpha (9–14 Hz), beta (15–30 Hz), and gamma (30–50 Hz). The fractional amplitude for each above conventional LFP sub-frequency band was calculated by first determining the mean amplitude for each band after FFT, and then dividing each of these mean values by the mean amplitude of the entire 1–50 Hz range.

#### 2.3.4 Resting state MRI data analysis

Preprocessing of resting-state MRI data was conducted using the AFNI software package (Cox and Hyde, 1997). Spatial independent component analysis (ICA) from the Melodic package (FSL software, Oxford University, UK) was used on each scan separately to identify and remove noisy components related to respiration and cardiac pulsation. Images from each session were coregistered to a common 3D anatomical space (Lu et al., 2010). The registered data were then subjected to slice-timing correction, linear and quadratic trend removal, and spatial smoothing with a Gaussian kernel (full width at half maximum (FWHM) = 0.6 mm). After preprocessing, wavelet transform (Luo et al., 2020) was used to calculate the fractional amplitude of low-frequency fluctuations (fALFF) (0.01–0.333 Hz) in four different frequency bands (0.01–0.027 Hz, 0.027–0.073 Hz, 0.073–0.198 Hz, and 0.198–0.333 Hz) (Zou et al., 2008; Zuo et al., 2010). The selected voxels (Figure 1) corresponded to the approximate location of the electrode tip, allowing direct comparison of the LFP and BOLD signals. Moreover, the LFP probe did not significantly interfere with the BOLD signal, as evidenced by intact contralateral connectivity from the affected striatum (Supplementary Figure 1).

#### 2.3.5 Paired T-test

Each rat underwent 6 pre- and 6 post-AMPA infusion sessions, resulting in 48 pairs (8 rats × 6 sessions) for the paired t-test analysis. To minimize selection bias, we permuted the pre- and post-infusion sessions across all rats and repeated the paired t-test 1000 times to generate average.

#### 2.3.6 Two-way repeated ANOVA

Two-way repeated measures ANOVA (frequency bands × pre- and post-infusion) was used to test the interaction effects of both LFP and BOLD signals.

## 3 Results

**FIGURE 2:**
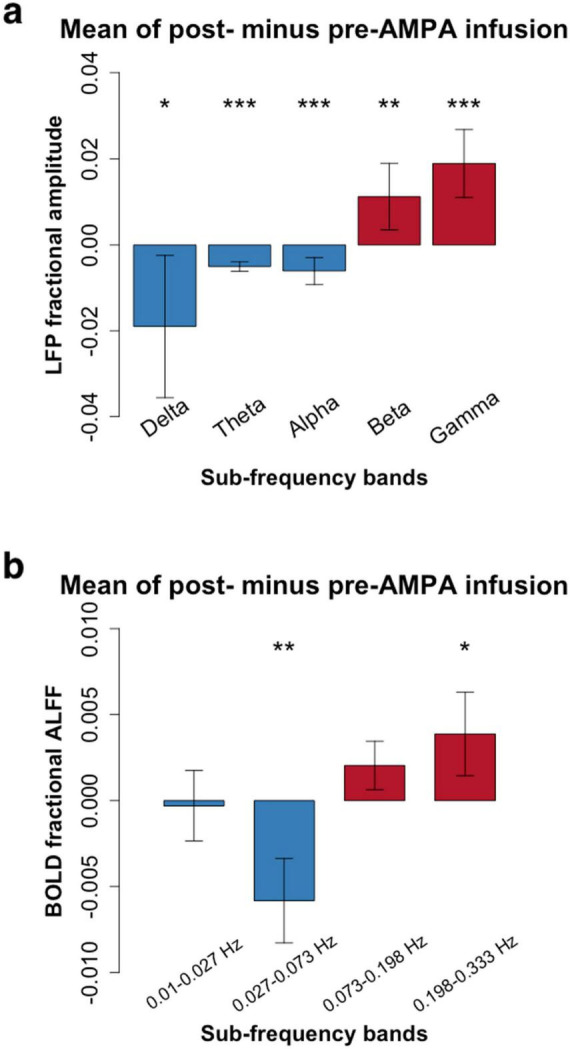
Frequency-dependent pharmacological manipulation effects (mean amplitude of post- minus pre-AMPA infusion). **(a)**, Local field potential (LFP) in 5 conventional frequency bands (Delta: 1–4 Hz; Theta: 5–8 Hz; Alpha: 9–14 Hz; Beta: 15–30 Hz; Gamma: 30–50 Hz). **(b)**, Resting-state functional magnetic resonance imaging (RS-fMRI) in 4 frequency bands (0.01–0.027 Hz; 0.027–0.073 Hz; 0.073–0.198 Hz; 0.198–0.333 Hz). Paired t-tests showed that the high frequency bands of both LFP (Beta and Gamma) and RS-fMRI (0.198–0.333 Hz) were significantly increased after AMPA infusion (*P < 0.05; **P < 0.01; ***P < 0.001. More details were shown in Tables 1, 2).

After AMPA infusion, we observed a decrease in amplitude within the low frequency bands and an increase in amplitude within the high frequency range for both LFP and RS-fMRI signals (Figure 2). Specifically, the amplitude of the BOLD signal exhibited a reduction in the low frequency band (0.027–0.073 Hz) but an elevation in the high frequency band (0.198–0.333 Hz) following AMPA infusion (Figure 2 and Tables 1, 2).

**TABLE 1:** LFP paired *T*-test results.

	*N*	Fractional amplitude		*t*	*p*
		pre	post		
Delta	48	0.444 ± 0.040	0.425 ± 0.064	−2.271	0.028*
Theta	48	0.174 ± 0.011	0.169 ± 0.012	−4.117	0.000***
Alpha	48	0.137 ± 0.013	0.131 ± 0.011	−3.961	0.000***
Beta	48	0.149 ± 0.016	0.160 ± 0.024	2.986	0.005**
Gamma	48	0.096 ± 0.012	0.114 ± 0.026	5.013	0.000***

All the LFP sub-frequency bands have significant differences, low sub-frequency bands (from Delta to Alpha) decrease the amplitude while high sub-frequency bands (Beta and Gamma) increase.

*P < 0.05;

**P < 0.01;

***P < 0.001.

**TABLE 2:** BOLD paired *T*-test results.

	*N*	Fractional ALFF		*t*	*p*
		Pre	Post		
freq1	48	0.082 ± 0.008	0.081 ± 0.009	−0.183	0.856
freq2	48	0.188 ± 0.010	0.182 ± 0.008	−3.111	0.004**
freq3	48	0.424 ± 0.007	0.426 ± 0.010	1.236	0.224
freq4	48	0.287 ± 0.009	0.291 ± 0.009	2.212	0.033*

Similar changes have shown at BOLD signal, low sub-frequency band (freq2) significantly increase the amplitude while the high sub-frequency band (freq4) decrease.

*P < 0.05;

**P < 0.01.

**FIGURE 3:**
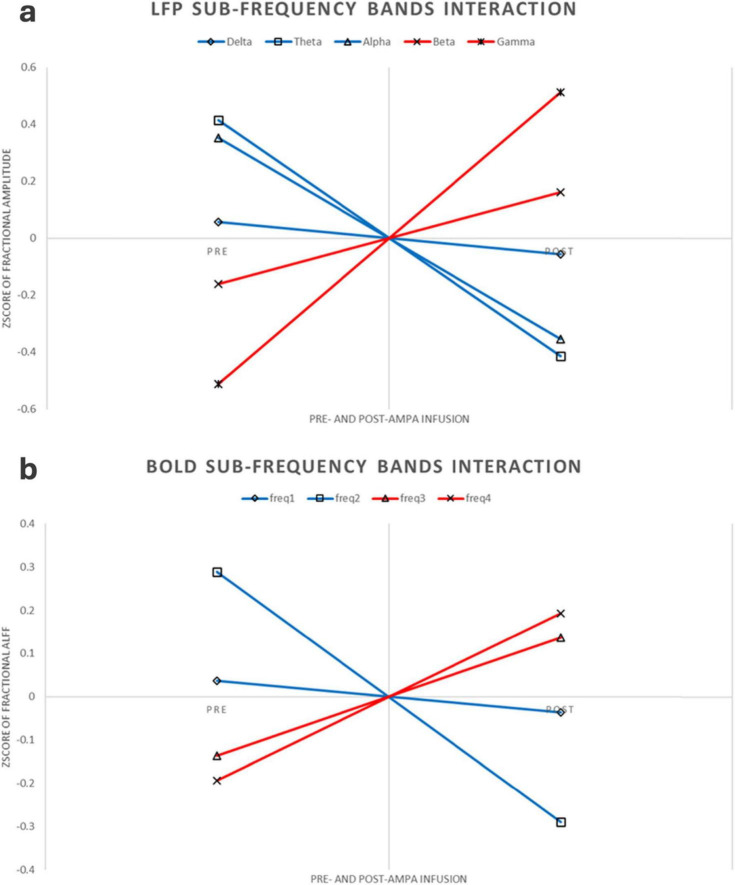
LFP and BOLD sub-frequency bands interaction. **(a)** LFP interaction pattern. For better comparison, the figure here were using Z-transform along each rat, increased or decreased sub-frequency bands were using different colors. **(b)** BOLD interaction pattern. Same as the LFP interaction, this figure was using Z-transform along each rat, increased or decreased sub-frequency bands were using different colors.

This experiment was a 2 × 2 repeated measures design. Repeated measures ANOVA showed that LFP and BOLD signal were significantly different in sub-frequency band, and the interaction effect of frequency band and AMPA modulation was significant (Tables 3, 4 and Figure 3).

**TABLE 3:** LFP fractional amplitude within-subjects contrasts.

	df	Mean square	*F*	sig
Frequency bands	1.135^+^	6.024^+^	1190.215	0.000***
AMPA infusion	1.000	0.000	2.391	0.129
AMPA infusion × Frequency bands	1.152^+^	0.019^+^	10.039	0.002**

There is significant interaction effect for different LFP sub-frequency bands. +: The Mauchly’s Test of Sphericity showed Chi-Square (Frequency bands) = 339.377 (*P* < 0.05), Chi-Square (AMPA infusion × Frequency bands) = 326.197 (*P* < 0.05), therefore, Greenhouse-Geisser calibration was selected for correction;

**P < 0.01;

***P < 0.001.

**TABLE 4:** BOLD fractional ALFF within-subjects contrasts.

	df	Mean square	*F*	sig
Frequency bands	1.941^+^	3.190^+^	18175.102	0.000***
AMPA infusion	1.000	0.000	0.409	0.526
AMPA infusion × Frequency bands	2.192^+^	0.001^+^	4.735	0.009**

There is significant interaction effect for different BOLD sub-frequency bands as well. +: The Mauchly’s Test of Sphericity showed Chi-Square (Frequency bands) = 36.451 (*P* < 0.05), Chi-Square (AMPA infusion × Frequency bands) = 24.362 (*P* < 0.05), therefore, Greenhouse-Geisser calibration was selected for correction;

**P < 0.01;

***P < 0.001.

## 4 Discussion

The majority of RS-fMRI studies have predominantly focused on low-frequency (< 0.08 Hz) signals in the context of brain disorders. However, a few RS-fMRI studies have reported abnormally increased amplitudes in the high-frequency band of the BOLD signal, accompanied by decreased amplitudes in the low-frequency band, in certain brain disorders (Malinen et al., 2010; Otti et al., 2013; Hong et al., 2013; Hong et al., 2018; Ries et al., 2019; Wang et al., 2020). For instance, two independent studies have shown increased RS-fMRI fluctuation amplitude in the high-frequency band along with decreased low-frequency amplitude in the anterior cingulate cortex and anterior insula of patients with chronic pain (Malinen et al., 2010; Otti et al., 2013). In the present study, we found that the increased amplitude in the high-frequency band, combined with a decreased amplitude in the low-frequency band of RS-fMRI, mirrors the frequency-dependent changes observed in LFP amplitudes (Figure 2). These findings suggest that increased high-frequency RS-fMRI amplitudes in brain disorders reflect abnormally heightened neuronal activity in high-frequency bands, which may be linked to underlying pathophysiology.

It is important to highlight the analytical approach used in the current study. Most existing studies of simultaneous LFP-fMRI recordings have focused on correlations between LFP power time series and RS-fMRI time series. In contrast, our study analyzed the amplitude of RS-fMRI signals (Zou et al., 2008; Zang et al., 2007) in a manner similar to the analysis of LFP amplitude (Birn, 2012). The amplitude of low-frequency fluctuation (ALFF) is one of the most widely used methods to depict local RS-fMRI activity (Di Martino et al., 2014). ALFF analysis involves examining the single time series within a specific brain region, which allows for precise localization of abnormal activity. The fALFF index is normalized in the frequency domain based on ALFF, thereby mitigating the impact of noise and enhancing the stability and comparability of the results (Zou et al., 2008). In this study, we analyzed not only the lower-frequency components but also the higher-frequency components of the RS-fMRI signal (Malinen et al., 2010; Otti et al., 2013; Hong et al., 2013; Hong et al., 2018; Ries et al., 2019; Wang et al., 2020).

The choice of the striatum for LFP recordings was informed by its central role in motor and cognitive functions, as well as its involvement in a wide range of neuropsychiatric disorders. This makes the striatum a relevant region for understanding frequency-dependent neuronal dynamics in the context of brain disorders. However, it remains unclear to what extent the findings can be generalized to other brain regions. Future studies should investigate whether similar frequency-dependent modulation patterns are present in other key brain areas, such as the prefrontal cortex, to better understand the broader applicability of these findings.

Several limitations should be acknowledged. First, the sample size in this study (*n* = 8) is relatively small. While the results are robust, this limitation may impact the statistical power and generalizability of the findings. Future studies should aim to include larger sample sizes to enhance the reliability of the conclusions. Additionally, we used a TR of 1.5 seconds, which limits the ability to analyze RS-fMRI signals above 0.34 Hz. Future studies should use faster scanning protocols to capture higher-frequency signals. Another limitation is that, due to magnetic susceptibility and subsequent signal loss at the thicker parts of the electrode (Figure 1), only the electrophysiological signal from the tip channel was analyzed. This constraint may have reduced the overall richness of the LFP data obtained.

Finally, the frequency-dependent modulation pattern observed in our study points to an intriguing mechanism of neuronal activity regulation. The increased high-frequency amplitude, along with decreased low-frequency amplitude, may reflect a shift towards heightened excitatory activity, potentially contributing to dysregulation in brain disorders. This could represent a compensatory mechanism or a maladaptive process linked to disease pathology. Future studies should aim to elucidate the underlying cellular and network mechanisms of this modulation, which may provide insights into the functional significance of these changes in the context of both health and disease.

## 5 Conclusion

In this study, we demonstrated a highly similar frequency-dependent pattern in the amplitude of RS-fMRI and LFP following pharmacological manipulation. We observed that the amplitudes of RS-fMRI and LFP signals covaried in a similar manner, with decreased low-frequency and increased high-frequency amplitudes. This finding reveals, for the first time, a potential direct coupling between high-frequency RS-fMRI signals and LFP activity. Furthermore, our results suggest that high-frequency RS-fMRI components, rather than low-frequency ones, may have significant physiological and pathophysiological relevance.

## Data Availability

The raw data supporting the conclusions of this article will be made available by the authors, without undue reservation.
