# Supplementary material for: Neuronal basis of high frequency fMRI fluctuation: direct evidence from simultaneous recording
Source: Front Hum Neurosci. 2024 Oct 31;18:1501310. doi: 10.3389/fnhum.2024.1501310 (PMC11560898; doi:10.3389/fnhum.2024.1501310)
Supplement: Supplementary file 1 [file Data_Sheet_1.docx]

**Supplementary material**

**
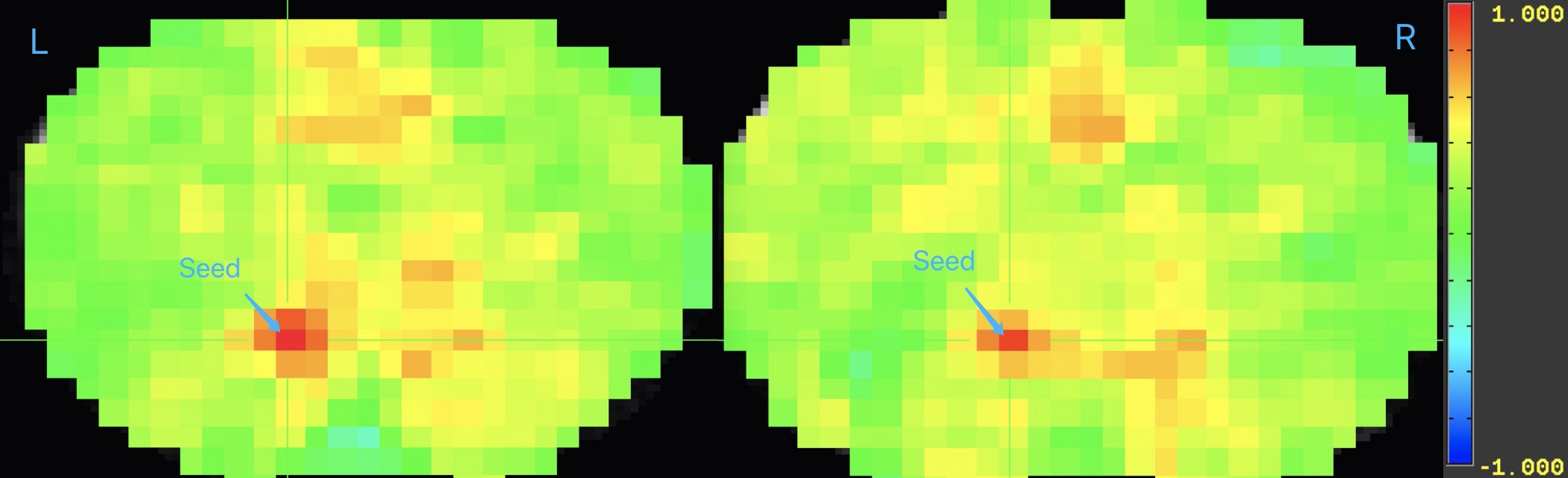
**

**SI Fig 1. Example for ROI (2 voxels) based connectivity.** We conducted a ROI based connectivity analysis on baseline sessions by using a ROI near the tip of the electrode as a seed to calculate its correlation with other voxels in the brain. The time series were filtered to conventional RS-fMRI band at 0.01-0.1 Hz for illustrating the spatial distribution.
